# Influence of auditory-based cognitive training on auditory resolution, executive function, and working memory skills in individuals with mild cognitive impairment – a pilot randomized controlled study

**DOI:** 10.12688/f1000research.152775.2

**Published:** 2025-01-13

**Authors:** Priya G, Kishan MM, VaniLakshmi R, Gopee Krishnan

**Affiliations:** 1 Department of Speech and Hearing, Manipal Academy of Higher Education, Manipal, Karnataka, 576104, India; 2 Department of Data Science, Prasanna School of Public Health, Manipal, Karnataka, 576104, India

**Keywords:** Mild cognitive impairment, Auditory processing, Cognitive training, older adults, memory.

## Abstract

**Background:**

Age-related central auditory processing disorder and mild cognitive impairment (MCI) can be concomitant in older adults, making it difficult to communicate, especially in challenging listening conditions. This preliminary study investigated the efficacy of auditory-based cognitive training on the auditory processing abilities and cognitive functions of older adults with MCI.

**Methods:**

In this randomized controlled trial twenty-two older adults with mild cognitive impairment (MCI) were randomly assigned to either an experimental (n=11) or a control group (n=11). The experimental group received 15 cognitive training sessions through tasks involving the auditory domain. The outcome measures of this study included auditory resolution (Temporal gap detection, frequency discrimination, and modulation detection) and cognitive measures (Trail making tests and digit recall), which were administered at three-time points (before training, post-training, and follow-up). The linear mixed model computed the effects of training on the outcome measures.

**Results:**

A significant improvement was observed in the modulation detection threshold between baseline and follow-up and between post-training and follow-up sessions. However, GDT and FD thresholds did not reveal any statistically significant difference. In the trail making test, Part B showed consistent significance across the time points, whereas Part A and the delayed recall task showed no significant difference.

**Conclusion:**

Auditory-based cognitive training may improve auditory processing and executive function in older adults with mild cognitive impairment (MCI).

**Trial registration:**

CTRI/2019/01/017073, registered on 14.01.2019

## Introduction

Aging is a natural process that affects people, causing a decline in cognitive and sensory abilities. ^1^ Aging leads to degenerative changes in the auditory structures and functions in older adults above 65 years, resulting in Presbycusis or Age-Related Hearing Loss (ARHL). Around 65% of adults above 60 experience this hearing loss, with the prevalence rate increasing from 15.4% among older adults above 60 to 58.2% among older adults above 90. ^2^ ARHL is caused by the loss of sensory cells and degenerative changes in the central auditory nervous system. ^3^ Another common condition that affects older adults is mild cognitive impairment (MCI). MCI is characterized by a decline in memory, attention, and cognitive function, ^4^ and it is a precursor of dementia, especially if it is an amnestic type. ^5^ ARHL makes it difficult to perceive speech, particularly in noisy environments, and can lead to a decline in cognitive function. ^6^^,^^7^ MCI can exacerbate speech perception problems. ^8^^,^^9^ Therapeutic management or training addressing sensory, cognitive, and learning issues may slow down or lessen the impact of aging.

Auditory and cognitive training are important components of rehabilitation for both healthy and aging populations, including those with disorders. Auditory training actively involves the trainees distinguishing the sounds presented systematically. ^10^ Although auditory training was initially used to enhance sensory refinement of speech sounds (bottom-up), literature shows it can also be beneficial for top-down processes, which are important for listening, especially in challenging conditions. ^11^^,^^12^ Cognitive training involves using standardized tasks that are cognitively challenging to improve an individual’s cognitive functions. ^13^ Studies have shown that cognitive interventions effectively enhance the cognitive function of older adults with Mild Cognitive Impairment (MCI). ^14^^–^^16^ They also improve auditory and cognitive functions, enhancing speech perception in various situations. ^17^^,^^18^

Many people have shown interest in exploring this type of training to alleviate cognitive and perceptual impairments. ^11^ In 2009, Smith conducted cognitive training sessions for older adults with normal cognitive function and observed improved auditory memory and attention. ^19^ Similarly, Yusof et al. (2019) examined the benefits of auditory-cognitive training among older adults with and without neurocognitive impairment. ^20^ Participants were trained in auditory perception, such as word and sentence recognition in noise, and auditory-cognitive tasks, such as word span, word order, and word position. Though both groups benefited from the training, improvement was more noticeable among individuals with normal cognitive functions in various domains. Similarly, in 2014 Avila and her colleagues studied the effectiveness of auditory training on the auditory and cognitive skills of older adults with Mild Cognitive Impairment. The training stimulated auditory skills such as auditory memory, selective attention, figure-ground, temporal processing, auditory closure, and binaural integration. The training resulted in the improvement of auditory skills but did not generalize to cognitive skills. ^21^

A recent study by Kawata et al. (2022) investigated the benefits of auditory and cognitive training in healthy older adults. ^22^ They provided auditory-cognitive training (ACT), auditory training (AT), and cognitive training (CT). The results indicated that the ACT group exhibited significant differences in brain structure changes compared to the other groups. It is worth noting that the study had a limitation in that it relied solely on pure tone audiometry as an auditory measure.

Murphy et al. (2011) conducted a study on the effectiveness of auditory training in an adult who had suffered a traumatic brain injury. The patient had difficulties with auditory processing and cognitive skills such as temporal processing, verbal memory, working memory, and verbal fluency. The auditory training not only enhanced auditory skills but also led to improvements in central processes (top-down processing), which consequently had a positive impact on cognitive abilities as well. Both auditory and cognitive training can strengthen neural pathways and circuits through training, leading to improved auditory perception and cognitive abilities. ^23^ Therefore, actively involving older adults in such training can bring about changes in the brain, ultimately enhancing their auditory and cognitive functions.

Numerous studies have demonstrated that cognitive interventions can enhance the quality of life in older adults with and without Mild Cognitive Impairment MCI. ^24^^–^^26^ However, there is a dearth of literature on auditory-based cognitive training, and the few existing studies have varying participant selection, training materials, and assessment measures. One study evaluated auditory-based cognitive measures alongside auditory cognitive training, but they used pure tone audiometry as an outcome measure, which is a peripheral test considered to be less influenced by cognitive function. ^20^ Therefore, the current study was designed to evaluate the impact of auditory-based cognitive training on auditory processing skills and cognitive abilities of older adults with cognitive impairment.

## Methods

### Study design and setting

This study was a pilot randomized controlled trial research, conducted at the Department of Speech and Hearing, Manipal College of Health Professions. This study included an experimental group which received cognitive training and a control group who did not receive any treatment. The assessment and training for all the participants were conducted at participants’ home in a silent environment. The procedures of this study were reviewed and approved by the Institutional Ethics Committee, Kasturba Hospital, Manipal (IEC 704/2017) on 15.11.2017, and the Clinical Trials Registry of India CTRI/2019/01/017073, registered on 14.01.2019.

### Participants

The participants were recruited from the community (Udupi and nearby districts, Karnataka) through geriatric camps, community visits and screening inmates from old age homes. This preliminary study included 22 older adults with mild cognitive impairment (MCI). The group consisted of 11 males and 11 females, with a mean age of 69.18 (6.35). All participants had a Montreal Cognitive Assessment (MoCA) score between 19 and 25 and had no history of neurological conditions or psychological disorders. The participants were randomly assigned to either the control group (n=11) or the experimental group (n=11). All participants had normal or corrected-to-normal vision in both eyes. They also had a mean (SD) pure tone average of 21.4 (3.61) dBHL in the right ear and 23.4 (4.84) dBHL in the left ear, as assessed across audiometric frequencies (250, 500, 1 kHz, 2 kHz, 4 kHz, and 8 kHz). All participants were native Kannada Speakers with proficiency in reading and writing and had completed at least ten years of education.

### Randomization and blinding

The participants were randomly assigned to either the experimental group or the control group using a simple randomization method involving the drawing of chits. The enrolled participants were asked to pick a chit and based on that they were assigned to either of the groups. The primary investigator provided intervention to all the participants in the experimental group, however all the three assessments (pre, post and follow up) were conducted by qualified audiologist and speech and language pathologists who were not a part of this study. The statistical analysis was performed by the primary investigator, but the interpretation was made after discussing with other investigators of this study who were unaware of the group allocation including a statistician.

### Participant groups

**Experimental group**

The group’s participants underwent 15 sessions of Auditory-based cognitive training (AbCT) using Smriti Shravan Software Version 2 (Kumar & Maruthy, 2013) ^27^ [ https://sites.google.com/u/0/s/1L2VuSNH1Y6DhrjkLs830TtvUhNjcfmUb/edit?usp=sites_home]. Appropriate permission was obtained for the usage of all the assessment tools, screening checklist and training software for this study. The training was provided to all the participants in a relatively silent environment at their residence based on their preferences. Each session lasted 60 minutes and included various tasks such as Forward Span, Backward Span, Running Span, Ascending Span, Descending Span, and Math Span tasks. The training module for each task consisted of digits from 1 to 9 presented through auditory modality, and the number of digits presented varied from 2 to 9. The stimuli were presented in the Kannada language, and the training was provided through the laptop via speakers set at a comfortable loudness level. Before initiating the training, the baseline of the above-mentioned tasks for all the participants were obtained. For each participant the difficulty level of the training was decided based on their baseline performance. All the participants listened carefully to the digits and repeated them verbally in the required order based on their task.

In the Forward Span task, participants repeated all the digits they heard in the same order as quickly as possible. In the Backward Span task, they repeated the digits heard in reverse order. The Running Span task used digits from 1 to 9, and participants recalled the last n digits of a set of numbers as quickly as possible, where n varied from 2 to 9. In the Ascending Span task, participants repeated the digits in ascending order, while in the Descending Span task, they repeated the digits in descending order. In the Math Span test, participants memorized and recalled sequences of digits ranging from 2 to 6 while solving simple arithmetic operations.

The difficulty level of each task was varied by increasing the number of digits from 2 to 9 based on their performance. To progress to the next level, participants had to achieve an accuracy score of 80%. If they did not meet this requirement, they continued training with the same task until they achieved a score of 80%. All participants’ data were included in the analysis since they completed 15 training sessions. Assessments were conducted at three time points - baseline, post-training (after 15 days of training), and follow-up (after one month of post training assessment).

**Control group**

The control group did not receive any intervention and was observed for the same duration as the experimental group. They remained passive throughout the study as they were not assigned any activity, unlike the training provided to the experimental group. Assessments were conducted for the control group also at three-time points – baseline, post observation (after 15 days of baseline assessment, follow-up observation (after 1 month of post observation assessment).

### Auditory resolution measures

The study used Angel Sound ^TM^, a free PC-based interactive listening rehabilitation program developed by Tiger Speech Technology, to measure individuals’ auditory resolution measures. The auditory resolution measure refers to an individual’s ability to detect the smallest variations in a given stimulus, such as variations in frequency (Hz) and time (dB or ms). This measure aimed to assess the listeners’ ability to distinguish subtle acoustic differences in spectral, temporal, and amplitude domains, which form the foundation for complex speech perception. The program uses a two-down/one-up adaptive method presented in a three-alternative-forced-choice paradigm. The adaptive run stops after 30 trials, with at least four reversals. The threshold is calculated as the average response across the reversals, achieving a 71% accuracy convergence.

The Frequency Discrimination (FD) test was conducted using three pure tones lasting 300 ms each, with a rise and fall time of 10 ms. These tones were separated by an interstimulus interval (ISI) of 500 ms. Two of the three tones had a frequency of 1000 Hz, considered the standard frequency, while one had a different frequency. The initial frequency difference between the standard and different frequencies was 25.6 Hz. The software adjusted the step size based on participant responses. It gradually increased the step size for incorrect responses and decreased it for correct responses.

The temporal modulation (MD) task involved three carrier stimuli consisting of broadband noise (BBN). Each stimulus lasted for 350 ms, and there was an inter-stimulus interval (ISI) of 500 ms. Participants were instructed to identify the stimulus that was different (modulated) from the other two stimuli. One of the three intervals was modulated at 10 Hz, whereas the other had no modulation. The modulation depth of the stimulus ranged from no modulation (No) to a minimum of 1% modulation depth (-40 dB) and a maximum of 100% modulation (0 dB). The software adopts a 1 dB step size for stimulus presentation.

The Gap Detection Task (GDT) consisted of three bursts of Broadband Noise (BBN) sounds, each lasting 250 ms, with a 500-ms pause between them. One of the three intervals contained a silent gap in the middle of the noise bursts, while the other two contained only noise bursts. The duration of the silent gap varied from trial to trial based on the participant’s response. The software adopts a step size of 1 ms (0 to 10 ms), 2 ms (10 to 20 ms), 5 ms (20 to 50 ms), 10 ms (50 to 100 ms), 20 ms (100 to 200 ms) and 50 ms (200 to 500 ms) respectively between the presentations.

The stimuli used in the test were played through a MAICO MA53 clinical audiometer calibrated beforehand via speakers in a quiet environment. The intensity of the sounds was adjusted to each participant’s most comfortable level (MCL).

### Cognitive measures

The study assessed the cognitive abilities of the participants using the Neurocognitive Toolbox (ICMR-NCTB), which The Indian Council of Medical Research developed. For this study, two tests from the toolbox were considered: the Trail Making Test (TMT) and the Verbal Learning Test. The TMT (Black & White) evaluates a person’s executive function through two segments: TMT Part A and TMT Part B. In TMT Part A, the participant must draw a line sequentially connecting 25 numbered circles on a page as quickly as possible. In TMT Part B, the participant must connect circles while alternating between numbers and circles in two colors, black and white. The TMT Part B is more challenging and takes longer time than TMT Part A. Both parts are timed, and the time taken to complete the task is recorded as the response. The difference in time to complete the task between Part A and Part B estimates the person’s executive function index (Part B-A).

**Figure 1.**
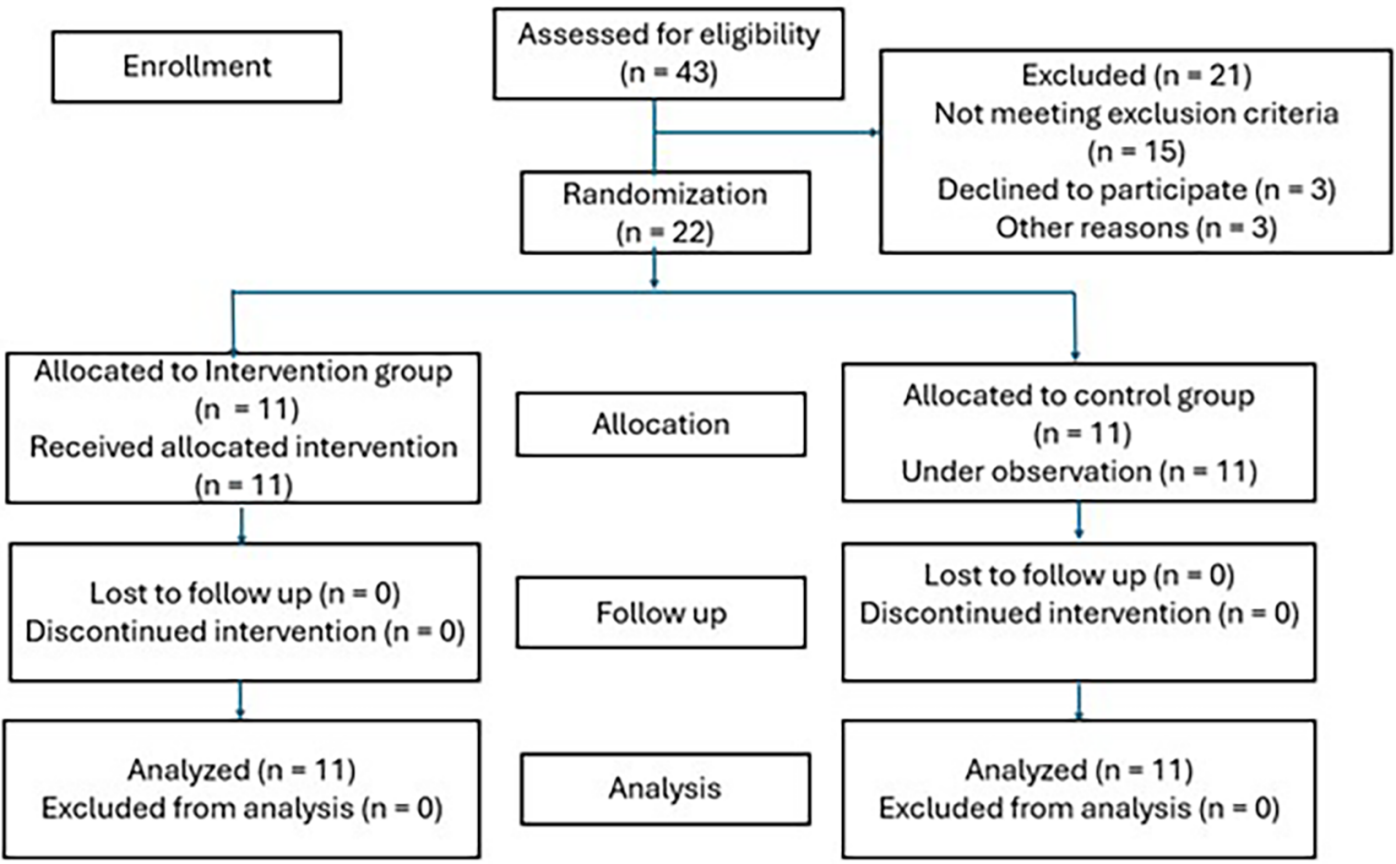
Participant flow diagram.

The Verbal Learning Test measures the participants’ episodic memory and records their immediate and delayed recall and recognition memory. The test presents a set of ten unrelated words orally to the participants over three trials. After each trial, the participants are asked to recall the words immediately in any order, and their responses are recorded. After 20 minutes, the participants are asked to recall the set of 10 words and identify the 10 words from a list of similar words. A diagram of participant flow is shown in Figure 1.

### Data analysis

Linear mixed model analyses were performed on the scores obtained during pre-, post, and follow-up performances. The dependent variables were the responses from Trail Making Tests, auditory resolution tasks, and delayed recall. We added fixed effects of cognitive assessment across time and group and the possible interaction between group and time. We used Jamovi software (version 2.3.28) to compute statistical data.

## Results

During the study period, out of 43 participants who were assessed for eligibility, 22 of them were enrolled in the study. All 11 participants in the experimental group completed the training, and their data was included in the analysis. The statistical analysis indicated no significant differences in baseline assessments between the control and experimental groups for most outcome measures, except for TMT Part B-A ( Table 1). Table 2 presents the mean and SD values for auditory resolution tasks, TMT, and DR outcome measures. The study found that AbCT was effective in three outcome measures: MD, TMT-B, and TMT B-A. The MD task showed a significant main effect of group versus time interaction (p=0.038). However, no significant main effect was observed for either group (p=0.497) or time (p=0.186). No significant main effect of time, group, or interaction between time and group was found for the FD and GDT tasks.

**Table 1.** Baseline demographic information.

Variables	Experimental group (n=11) Mean (SD)	Control group (n=11) Mean (SD)	p-value
Age (years)	70.4 (5.71)	68.0 (7.18)	0.403
MoCA	22.2 (1.99)	21.5 (2.16)	0.481
FD	129.36 (119.20)	56.45 (41.45)	0.070
MD	-13.75 (2.32)	-14.02 (3.83)	0.847
TGD	22.71 (42.48)	9.65 (3.88)	0.322
TMT Part A	98.27 (46.29)	99.27 (29.15)	0.952
TMT Part B	289.73 (120.36)	217.73 (47.82)	0.080
TMT Part B-A	191.45 (80.71)	118.45 (33.09)	0.012 *
DR	4.55 (3.45)	5.55 (2.30)	0.433

* Level of Significance ( *p*<0.05).

**Table 2.** Statistical results from linear mixed model along with Mean (standard deviation) values for auditory and cognitive measures of both the groups.

	Experimental Group			Control group			F	P value
	Baseline	Post training	Follow-up	Baseline	Post training	Follow-up		
	Mean (SD)	Mean (SD)	Mean (SD)	Mean (SD)	Mean (SD)	Mean (SD)		
**FD**	129.36(119.20)	105.95 (82.24)	67.25 (74.02)	56.45 (41.45)	53.97 (47.09)	67.76 (53.96)	2.79	0.096
**MD** ^**b**^ ^**c**^	-13.75 (2.32)	-13.15 (4.61)	-15.77 (1.71)	-14.02 (3.83)	-16.13 (2.40)	-14.76 (3.55)	3.58	0.038
**TGD**	22.71 (42.48)	10.29 (6.11)	7.15 (2.23)	9.65 (3.88)	8.20 (3.45)	8.00 (3.80)	0.943	0.346
**TMT Part A**	98.27 (46.29)	93.00 (35.13)	76.45 (27.58)	99.27 (29.15)	87.64 (37.39)	95.36 (45.93)	1.700	0.196
**TMT Part B** ^**a**^ ^**c**^	289.73(120.36)	225.36 (82.36)	221.91 (89.53)	217.73 (47.82)	209.18 (84.46)	219.00 (84.80)	4.161	0.023
**TMT B-A** ^**a**^ ^**c**^	191.45 (80.71)	132.36 (56.36)	145.45 (64.85)	118.45 (33.09)	121.55 (51.30)	123.64 (60.34)	4.20	0.023
**DR**	4.55 (3.45)	5.45 (3.70)	6.91 (2.77)	5.55 (2.30)	6.36 (2.58)	7.27 (2.49)	0.562	0.575

F – F value

p – p value (Level of Significance <0.05).

^a^ Significant between baseline and post-training.

^b^ Significant between post-training and follow-up.

^c^ Significant between baseline and follow-up.

For the TMT-A task, there were no significant differences in time (p=0.164), group (p=0.733), and the interaction between group and time (p=0.196). However, significant main effects of time and group versus time interaction were observed in Part B and Part B-A, respectively, while the group effect was insignificant. There was a significant difference in the delayed recall tasks over time (p<0.001), but not between groups (p=0.525) or group versus time (p=0.575).

Post hoc analysis revealed a significant score difference between baseline-follow-up and post-follow-up in the Modulation Detection task. Furthermore, TMT-B and TMT B-A had statistically significant differences (p=0.023) during the TMT in the baseline-post and baseline-follow-up conditions.

## Discussion

This study aimed to investigate the benefits of AbCT in improving auditory processing and cognitive functions among older adults with mild cognitive impairment. The results revealed that AbCT positively impacted MD function, but no significant improvements were observed in FD and GDT. Similarly, TMT-B (executive function) showed significant differences compared to TMT-A and DR.

The positive impact of AbCT can be explained by two mechanisms involved in auditory processing. The first mechanism is the neurocognitive mechanism of acoustic signals, which discriminates and recognizes specific functions. The second mechanism is the attentional process, involving phenomena such as attention and memory. ^23^ Anderson et al. (2013) conducted a study to evaluate the effect of auditory training with six modules designed to increase the speed and accuracy of auditory processing cognitive training on the temporal precision of subcortical speech processing in noise using frequency following response (FFR). ^17^ They observed that the trained group exhibited faster neural timing and experienced improvements in memory, processing speed, and speech-in-noise perception compared to the control group. Song et al. (2012) investigated training-related malleability using a program that included cognitive based listening exercises to enhance speech-in-noise perception. ^28^ The trained individuals showed significant improvements in speech-in-noise perception, sustained even after six months of training. The subcortical responses in noise showed enhancements in the encoding of pitch-related cues, particularly for the time-varying portion of the syllable most susceptible to perceptual disruption. Similarly, Carcagno and Plack (2011) observed that the robustness of FFR neural phase locking to the sound envelope increased significantly more in trained individuals (pitch contour training) than in the control group. ^29^ These studies support the neurocognitive process and suggest that auditory cognitive training, or AbCT, can improve auditory processing.

Attentional processes and working memory are crucial in speech perception, especially in the presence of background noise. A study by Wong et al. (2010) found that activity in the prefrontal cortex and regions associated with memory and attention in speech perception in noisy environments increased, indicating that cognitive compensation may play a crucial role in aiding hearing in noisy environments and that the recruitment of general cognitive areas accompanies declines in sensory processing. ^30^ Similarly, O’Brien et al. (2017) provided auditory cognitive training (Brain fitness) to healthy older adults and observed that a P3b event-related potentials amplitude, latency, and P1-N1-P2 complex significantly improved post-training. However, no advantage was found in auditory perceptual processing. ^31^ Studies have shown that P3b originates from temporal-parietal activity associated with attention and appears related to subsequent memory processing. ^32^ Therefore, our study emphasized that AbCT could improve the efficiency of attention allocation and working memory, which might explain the training-related changes in the auditory processing and neurocognitive processes.

The differences in performance between the GDT and TMTF tests may be due to the sensitivity and reliability of the training and testing methods. A study by Yusof et al. (2019) investigated the impact of auditory-cognitive training on older adults with normal cognitive function and neurocognitive impairment. ^20^ They found that there was an improvement in some auditory measures such as HINT (quiet), GIN, PPST (humming), and DDT but not in HINT (Composite) and PPST (verbal) for both normal cognitive (NC) and neurocognitive impairment (NCI) groups. Additionally, they observed that the NC group showed more significant improvements than the NCI group, suggesting a higher potential for learning among NC participants. Furthermore, Shen (2014) reported no correlation between GDT and TMTF, and that age and hearing status had no impact on the tests. However, TMTF sensitivity improved as hearing thresholds decreased and worsened with age. ^33^

The other finding of our study revealed that the AbCT training positively impacted central executive function and attention, as indicated by a significant improvement in TMT-B and TMT B-A, while TMT-A and DR remained unaffected. This suggests that the training primarily influenced cognitive abilities related to attention and executive function rather than psychomotor skills or working memory. This observation agrees with the study by Kawata et al. (2022). They compared four groups - an auditory cognitive training group, an auditory training group, a cognitive training group, and a control group. The results showed that the auditory cognitive training group demonstrated more significant changes in regional gray matter volume in several brain regions than the other groups. The auditory training group significantly improved auditory measures and increased regional gray matter volume and functional connectivity (FC) in the left temporal pole. ^22^ These observations suggest there exists a functional connectivity between the auditory and cognitive processes. Therefore, auditory and cognitive training can improve cognitive and auditory skills in healthy older adults. However, they used a pure tone audiometer as an outcome measure that was less influenced by cognitive and auditory training. A study by Ruscheweyh et al. (2013) reported that executive function is related to regional gray matter volume, also a biomarker, in healthy older individuals. ^34^^,^^35^ These studies justify the improvement in executive function. Given the significant differences observed at baseline for TMT Part B-A, post training significance should be interpreted cautiously before generalizing. No significant difference in digit recall could be attributed to the persistence of benefits for speed of processing/auditory processing but not for memory, which is consistent with the findings of Borella et al. (2010), who showed that older adults maintained training enhancements for fluid intelligence and speed of processing but not for memory. ^36^

The current study results evaluated the potential of AbCT on the auditory and cognitive skills of older adults with MCI. However, these findings may not be generalized to a wider population due to several concerns. First, the relatively smaller sample size and the absence of electrophysiological tests, which are considered the highest level of evidence for assessing training-related benefits. Second, the characteristics of the participants involved in the study, where recruitment was based on MoCA screening rather than clinical diagnosis of MCI, may have influenced the results. This is mainly due to challenges in obtaining consent from the participants to undergo detailed cognitive and audiological assessment due to practical issues, less motivation, and family consent. Additionally, the number and duration of the training sessions were determined based on a literature search. An interventional study recommended an optimal dose of 12 to 14 sessions for cognitive training and 15 to 20 sessions for multidomain training. Variations in the individual characteristics of the participants were also observed in the study, which directly impacted the optimal dose and dose-response functions. ^37^

The secondary concern is that the heterogeneity of the MCI population makes it challenging to apply the findings broadly. Though all the participants were recruited based on the cut off score of MCI, their performance on the auditory, cognitive and baseline assessment before training were highly variable. Participants in this study performed slightly poorer in digit span tasks (forward and backward) when compared to a normative data obtained from older adults with MCI. ^38^ Including an active control group in this study would have eliminated the possibility of a placebo effect in the experimental group. Additionally, various factors, such as participant motivation and health condition on the day of assessment, could have influenced their performance. Furthermore, the literature supports the absence of evidence indicating differences between the active and passive control groups in cognitive interventions. ^39^ We also observed significant variability in the baseline data of the experimental and control groups, which we tried addressing using linear mixed models for statistical analysis, accounting for unbalanced data. As this was a preliminary study, future randomized controlled trials (RCTs) must consider these variations and address these limitations.

## Conclusion

This current preliminary study suggests that AbCT seems to restore age-related deficits in temporal processing in the brain, promoting better cognitive and perceptual skills, particularly in older adults with MCI. The results also suggest that auditory temporal skills could be adaptable and can be a favorable prognostic indicator. However, further research in this area is warranted with a large sample size to determine whether such auditory cognitive training can change auditory and cognitive functions.

## Ethics and consent

This study protocol was reviewed and approved by the Institutional Ethics Committee, Kasturba Hospital, Manipal (IEC 704/2017) on 15.11.2017, and the Clinical Trials Registry of India (CTRI/2019/01/017073). Written informed consent was obtained from all the participants who participated in this study.

## Data Availability

Name of the repository: Open Science Framework Project Title: Influence of Auditory-Based Cognitive Training on Auditory Resolution, Executive function, and Working Memory Skills in individuals with Mild Cognitive Impairment – A Preliminary study, DOI: 10.17605/OSF.IO/3J67R. ^40^ This project contains the following underlying data:
• Data for repository.xlsx • Clinical Trial Protocol.pdf Data are available under the terms of the Creative Commons Zero “No rights reserved” data waiver (CC0 1.0 Public domain dedication). Name of the repository: Open Science Framework Project Title: Influence of Auditory-Based Cognitive Training on Auditory Resolution, Executive function, and Working Memory Skills in individuals with Mild Cognitive Impairment – A Preliminary study, DOI: 10.17605/OSF.IO/3J67R. ^40^ This project contains the following underlying data:
• CONSORT 2010 Checklist & Flowchart.pdf Data are available under the terms of the Creative Commons Zero “No rights reserved” data waiver (CC0 1.0 Public domain dedication).

## References

[1] Deal JA Betz J Yaffe K : Hearing impairment and incident dementia and cognitive decline in older adults: the health ABC study. *J. Gerontol. A Biol. Sci. Med. Sci.* 2017;72(5):703–709. 27071780 10.1093/gerona/glw069 PMC5964742

[2] World Health Organization: *World report on hearing.* World Health Organization;2021.

[3] Jayakody DM Friedland PL Martins RN : Impact of aging on the auditory system and related cognitive functions: a narrative review. *Front. Neurosci.* 2018;12:125. 10.3389/fnins.2018.00125 29556173 PMC5844959

[4] Eshkoor SA Hamid TA Mun CY : Mild cognitive impairment and its management in older people. *Clin. Interv. Aging.* 2015;10:687–693. 10.2147/CIA.S73922 25914527 PMC4401355

[5] Janoutová J Serý O Hosák L : Is mild cognitive impairment a precursor of Alzheimer’s disease? Short review. *Cent. Eur. J. Public Health.* 2015;23(4):365–367. 10.21101/cejph.a4414 26841152

[6] Kawata NY Nouchi R Saito T : Subjective hearing handicap is associated with processing speed and visuospatial performance in older adults without severe hearing handicap. *Exp. Gerontol.* 2021;156:111614. 10.1016/j.exger.2021.111614 34728338

[7] Shechter Shvartzman L Lavie L Banai K : Speech perception in older adults: an interplay of hearing, cognition, and learning? *Front. Psychol.* 2022;13:816864. 10.3389/fpsyg.2022.816864 35250748 PMC8891456

[8] Bisogno A Scarpa A Di Girolamo S : Hearing loss and cognitive impairment: epidemiology, common pathophysiological findings, and treatment considerations. *Life.* 2021;11(10):1102. 10.3390/life11101102 34685474 PMC8538578

[9] McClannahan KS Mainardi A Luor A : Spoken word recognition in listeners with mild dementia symptoms. *J. Alzheimers Dis.* 2022;90(2):749–759. 10.3233/JAD-215606 36189586 PMC9885492

[10] Schow RL Nerbonne MA : Introduction to audiologic rehabilitation. *(No Title).* 2002.

[11] Pichora-Fuller MK Levitt H : Speech comprehension training and auditory and cognitive processing in older adults. 2012. 10.1044/1059-0889(2012/12-0025) 23233521

[12] Ferguson MA Henshaw H : Auditory training can improve working memory, attention, and communication in adverse conditions for adults with hearing loss. *Front. Psychol.* 2015;6:137455. 10.3389/fpsyg.2015.00556 PMC4447061 26074826

[13] Lampit A Hallock H Valenzuela M : Computerized cognitive training in cognitively healthy older adults: a systematic review and meta-analysis of effect modifiers. *PLoS Med.* 2014;11(11):e1001756. 10.1371/journal.pmed.1001756 25405755 PMC4236015

[14] He W Wang M Jiang L : Cognitive interventions for mild cognitive impairment and dementia: an overview of systematic reviews. *Complement. Ther. Med.* 2019;47:102199. 10.1016/j.ctim.2019.102199 31780033

[15] Zhang H Huntley J Bhome R : Effect of computerised cognitive training on cognitive outcomes in mild cognitive impairment: a systematic review and meta-analysis. *BMJ Open.* 2019;9(8):e027062. 10.1136/bmjopen-2018-027062 31427316 PMC6701629

[16] Chan AT Ip RT Tran JY : Computerized cognitive training for memory functions in mild cognitive impairment or dementia: a systematic review and meta-analysis. *NPJ Digit. Med.* 2024;7(1):1. 10.1038/s41746-023-00987-5 38172429 PMC10764827

[17] Anderson S White-Schwoch T Parbery-Clarka A : Reversal of age-related neural timing delays with training. *Proc. Natl. Acad. Sci. USA.* 2013;110:4357–4362. 10.1073/pnas.1213555110 23401541 PMC3600492

[18] Athilingam P Edwards JD Valdes EG : Computerized auditory cognitive training to improve cognition and functional outcomes in patients with heart failure: results of a pilot study. *Heart Lung.* 2015;44:120–128. 10.1016/j.hrtlng.2014.12.004 25592205

[19] Smith GE Housen P Yaffe K : A cognitive training program based on principles of brain plasticity: results from the Improvement in Memory with Plasticity-based Adaptive Cognitive Training (IMPACT) Study. *J. Am. Geriatr. Soc.* 2009;57(4):594–603. 10.1111/j.1532-5415.2008.02167.x 19220558 PMC4169294

[20] Yusof Y Mukari SZMS Dzulkifli MA : Efficacy of a newly developed auditory–cognitive training system on speech recognition, central auditory processing and cognitive ability among older adults with normal cognition and with neurocognitive impairment. *Geriatr. Gerontol. Int.* 2019;19(8):768–773. 10.1111/ggi.13710 31237107

[21] Ávila RRDA Murphy CFB Schochat E : Effects of auditory training in elderly with mild cognitive impairment. *Psicologia: Reflexão e Crítica.* 2014;27:547–555.

[22] Kawata NY Nouchi R Oba K : Auditory cognitive training improves brain plasticity in healthy older adults: Evidence from a randomized controlled trial. *Front. Aging Neurosci.* 2022;14:826672. 10.3389/fnagi.2022.826672 35431898 PMC9010026

[23] Murphy CFB Fillippini R Palma D : Auditory training and cognitive functioning in adult with traumatic brain injury. *Clinics.* 2011;66:713–715. 10.1590/S1807-59322011000400030 21655770 PMC3093805

[24] Hill NT Mowszowski L Naismith SL : Computerized cognitive training in older adults with mild cognitive impairment or dementia: a systematic review and meta-analysis. *Am. J. Psychiatry.* 2017;174(4):329–340. 10.1176/appi.ajp.2016.16030360 27838936

[25] Yun S Ryu S : The effects of cognitive-based interventions in older adults: a systematic review and meta-analysis. *Iran. J. Public Health.* 2022;51(1):1–11. 10.18502/ijph.v51i1.8286 35223620 PMC8837877

[26] Li F Parsons J Peri K : Effects of cognitive interventions on quality of life among adults with mild cognitive impairment: A systematic review and meta-analysis of randomised controlled trials. *Geriatr. Nurs.* 2022;47:23–34. 10.1016/j.gerinurse.2022.06.009 35816984

[27] Kumar AU Maruthy S : *Auditory cognitive training module.* Mysore: ARF funded departmental project submitted to All India Institute of Speech and Hearing;2013.

[28] Song JH Skoe E Banai K : Training to improve hearing speech in noise: biological mechanisms. *Cereb. Cortex.* 2012;22(5):1180–1190. 10.1093/cercor/bhr196 21799207 PMC3450924

[29] Carcagno S Plack CJ : Subcortical plasticity following perceptual learning in a pitch discrimination task. *J. Assoc. Res. Otolaryngol.* 2011;12:89–100. 10.1007/s10162-010-0236-1 20878201 PMC3015031

[30] Wong PC Ettlinger M Sheppard JP : Neuroanatomical characteristics and speech perception in noise in older adults. *Ear Hear.* 2010;31(4):471–479. 10.1097/AUD.0b013e3181d709c2 20588117 PMC2919052

[31] O’Brien JL Lister JJ Fausto BA : Cognitive training enhances auditory attention efficiency in older adults. *Front. Aging Neurosci.* 2017;9:322. 10.3389/fnagi.2017.00322 29046634 PMC5632656

[32] Polich J : Updating P300: an integrative theory of P3a and P3b. *Clin. Neurophysiol.* 2007;118(10):2128–2148. 10.1016/j.clinph.2007.04.019 17573239 PMC2715154

[33] Shen Y : Gap detection and temporal modulation transfer function as behavioral estimates of auditory temporal acuity using band-limited stimuli in young and older adults. *J. Speech Lang. Hear. Res.* 2014;57(6):2280–2292. 10.1044/2014_JSLHR-H-13-0276 25087722 PMC4372392

[34] Ruscheweyh R Deppe M Lohmann H : Executive performance is related to regional gray matter volume in healthy older individuals. *Hum. Brain Mapp.* 2013;34(12):3333–3346. 10.1002/hbm.22146 22815223 PMC6869861

[35] Cristofori I Zhong W Chau A : White and gray matter contributions to executive function recovery after traumatic brain injury. *Neurology.* 2015;84(14):1394–1401. 10.1212/WNL.0000000000001446 25746558 PMC4395886

[36] Borella E Carretti B Riboldi F : Working memory training in older adults: evidence of transfer and maintenance effects. *Psychol. Aging.* 2010;25(4):767–778. 10.1037/a0020683 20973604

[37] Belleville S Cloutier S Mellah S : Is more always better? Dose effect in a multidomain intervention in older adults at risk of dementia. *Alzheimers Dement.* 2022;18(11):2140–2150. 10.1002/alz.12544 35049127 PMC9786573

[38] Tripathi R Kumar K Bharath S : Indian older adults and the digit span A preliminary report. *Dement. Neuropsychol.* 2019;13:111–115. 10.1590/1980-57642018dn13-010013 31073387 PMC6497020

[39] Au J Gibson BC Bunarjo K : Quantifying the difference between active and passive control groups in cognitive interventions using two meta-analytical approaches. *J. Cogn. Enhanc.* 2020;4:192–210. 10.1007/s41465-020-00164-6 34337311 PMC8320766

[40] Priya G Krishnan G Kishan MM : Influence of Auditory-Based Cognitive Training on Auditory Resolution, Executive function, and Working Memory Skills in individuals with Mild Cognitive Impairment – A Preliminary study. 2024. 10.17605/OSF.IO/3J67R PMC11966092 40183007

