# Supplementary material for: Influence of auditory-based cognitive training on auditory resolution, executive function, and working memory skills in individuals with mild cognitive impairment – a pilot randomized controlled study
Source: F1000Res. 2025 Jan 13;13:1022. Originally published 2024 Sep 9. [Version 2] doi: 10.12688/f1000research.152775.2 (PMC11966092; doi:10.12688/f1000research.152775.2)
Supplement: Peer review history

# Reviewer report: Reviewer response for version 2

**DOI:** 10.5256/f1000research.176423.r370402

**Published:** 2025-04-07

**Authors:** Dr Pinki Singh

**Affiliations:** 1 NSCB Medical college, Jabalpur, India

In this study, impact of auditory cognitive training has been studied for the rehabilitation of healthy and aging population with MCI in terms of facilitating auditory processing skills & cognitive abilities.

 It has been observed that author has incorporated the changes suggested by previous reviewers.

 Introduction:  

- Well  written but as per the recent researches it has been found that MCI coexist with ARHL, so study suggesting the significance of cognitive training via auditory modality helps in managing MCI could have been added.

Methods:

Randomization & Blinding- Three assessment (pre, post & follow up) timings have been specified after review but it is required to mention the criteria’s of audiologist & SLP like,-1. fluency in specific language which will be required for instruction, 2. brief training was provided to them  to conduct recommended tests , 3. About minimum years of clinical experience.

Participant group-  - how 15 sessions were scheduled, was it scheduled daily, alternate or some other pattern of interval…

- Screening checklist- are authors talking about MoCA OR some other checklist? Found difficult to interpret.
- Training- was it provided at home to every participants? For every sessions? By primary investigators? If not then please specify the details. It is difficult for primary investigators to observe 11+11 participants individually for 15 days at participant’s home situation.
- Items: Items for the following tasks e.g. forward span, backward span, running span, ascending & descending span & math span tasks should be attached in appendix for better comprehensibility.
- Training to participants were provided before performing the tasks? Was there any practice trial/items for the respective tasks?
- Difficulty level has been mentioned throughout i.e. participant’s difficulties level was increased but nowhere difficulties level has been specified. E.g. difficulty level 1.: 3 digits stimulus was given/1-2 repetition of digits were done etc.  Difficulty level 3: 5 digits tasks were given , no repetition of tasks etc. has to be mentioned otherwise this study could not be generalized. It is required because as it has been stated “to progress to the next level, participant had to achieve an accuracy score of 80%...…”
- During the tasks, participants were provided with paper & pen/pencil or only mental task was allowed.
- Was there any sequence of tasks presentation to avoid any familiarity or adaptation effects?

Results:

 Above mentioned changes could be helpful for result section for better comprehensibility in terms of sequence of presentation of tasks and difficulties level.

 In table 1: In footnote, abbreviation’s extended form should be mentioned for “column A Variables”

 Discussion: it is well written, future directions could be added. It will be helpful for other researchers as well.

Is the work clearly and accurately presented and does it cite the current literature?

Yes

If applicable, is the statistical analysis and its interpretation appropriate?

Partly

Are all the source data underlying the results available to ensure full reproducibility?

Yes

Is the study design appropriate and is the work technically sound?

Partly

Are the conclusions drawn adequately supported by the results?

Partly

Are sufficient details of methods and analysis provided to allow replication by others?

Partly

Reviewer Expertise:

Cognitive disorders, Neuro Speech & Language Disorders

I confirm that I have read this submission and believe that I have an appropriate level of expertise to confirm that it is of an acceptable scientific standard, however I have significant reservations, as outlined above.

# Reviewer report: Reviewer response for version 2

**DOI:** 10.5256/f1000research.176423.r370406

**Published:** 2025-04-02

**Authors:** Sangamanatha Ankmnal Veeranna

**Affiliations:** 1 College of Nursing and Health Professions, School of Speech and Hearing Sciences, J.B. George Building, University of Southern Mississippi, Hattiesburg, Mississippi, USA

Abbreviations are often used in manuscripts to simplify the presentation of frequently repeated terms. However, it is important to use them consistently to prevent confusion. To enhance clarity, abbreviations should be spelled out the first time they appear, followed by the abbreviation in parentheses.

 Throughout the manuscript, there is a lack of consistency in using abbreviations. Several terms have been abbreviated, but not always in a consistent manner. For example, the abbreviation "MD" is used for temporal modulation tasks, but this term is not clearly defined or consistently applied throughout the manuscript.

 In the discussion, the abbreviation "TMTF" is introduced without any prior explanation, making it unclear to the reader what it represents. Additionally, the abbreviation "DR" is used multiple times throughout the manuscript, but its meaning is not explained. The abbreviation "TGD" is also mentioned in the result table (Table 2), but its definition is not provided, leaving the reader confused about its meaning. I assume that it is Temporal Gap Detection. Moreover, the abbreviation "MCI" is used inconsistently alongside the full term "Mild Cognitive Impairment."

 To improve readability and clarity, the authors should consider removing most of these abbreviations, especially those that are not commonly used or that could easily be understood without abbreviation. The manuscript would benefit from more careful attention to consistent terminology, with each abbreviation clearly defined upon its first use.

 Introduction:

 Paragraph 3 in the introduction: The authors need to maintain consistency when describing the study findings. For instance, the explanation of Smith’s (2009) study lacks clarity, while other studies are described in detail. This paragraph should be rewritten to improve its clarity and flow, providing more concise and coherent information. Additionally, the findings from Kawata et al. are not clearly presented, which further detracts from the overall understanding of the study.

 Last Paragraph in the introduction: The authors mention that there are numerous studies yet only cite three. This statement should be rephrased to better reflect the intended message. The paragraph seems to emphasize the need for study but lacks strength. The authors should revise this section to strengthen the rationale behind the research. It would be helpful to clearly explain why this study is being conducted. Moreover, much of the relevant literature is discussed in the manuscript's discussion section, rather than being appropriately included in the introduction. The authors should ensure that key studies are introduced earlier in the manuscript to set a stronger foundation for the research.

 Method:

 There is no clear explanation of how individuals with Mild Cognitive Impairment were identified and diagnosed. Was the diagnosis solely based on the Montreal Cognitive Assessment? If so, how was the Montreal Cognitive Assessment administered? Was it translated into Kannada for the participants? More information on the diagnostic criteria and procedures would help clarify this point.

 The authors calculated the hearing threshold average for all frequencies, but typically, averages are calculated for either three (0.5, 1, and 2 kHz) or four frequencies (0.5, 1, 2, and 4 kHz). It would be helpful for the authors to justify their decision to use all frequencies in this study, as it deviates from standard practice.

 The "Auditory Resolution measures" used by the authors lack proper justification. Why were these specific measures selected, and not others? Are these measures particularly relevant to auditory perception? Do they have a greater likelihood of showing changes with cognitive training compared to other measures? Additionally, it would be useful to know if previous studies have used similar auditory resolution measures and whether they have been effective in assessing auditory perception in populations with cognitive impairment.

 Regarding the psychoacoustic testing, were participants given any training prior to the tests? This detail is crucial to understand whether the participants were familiar with the testing procedures and to assess the validity of the results.

 For the frequency discrimination task, what was the step size used?

 Furthermore, were stimuli presented in a free-field setup? There is no information provided on how the calibration was performed, which is a critical aspect of ensuring the reliability and replicability of the study. Clear calibration procedures should be outlined to allow other researchers to replicate this study accurately. How was the most comfortable level determined?

 Providing more information on these aspects would significantly strengthen the manuscript and enhance the clarity and transparency of the methodology.

 Results:

 The statistical analysis is difficult to follow. It is unclear how the linear mixed model was conducted. The authors should explain the specific factors and covariates included in the model, as well as any interactions considered. Furthermore, there is no justification for the sample size, which is important for assessing the statistical power of the analysis. Providing more detailed information about the statistical methods and sample size would improve the clarity of the results.

 Discussion:

 The discussion section needs to be reorganized for better clarity and flow. Currently, it is difficult to follow, and the focus is more on previous research findings than on the implications of the current study’s findings. To improve readability, the section would benefit from subsections that guide the reader through the key points.

 While referencing past studies is essential for providing context, the primary focus should be on the current study's findings and implications. The authors should emphasize how their results contribute to the existing body of knowledge, and how they support or challenge previous research. Addressing any discrepancies or gaps in the literature will offer a clearer understanding of the study’s significance and enhance its relevance to the field.

 The authors will provide a more coherent discussion by shifting the focus to the study’s specific findings and their broader implications.

Is the work clearly and accurately presented and does it cite the current literature?

Partly

If applicable, is the statistical analysis and its interpretation appropriate?

Partly

Are all the source data underlying the results available to ensure full reproducibility?

Partly

Is the study design appropriate and is the work technically sound?

Yes

Are the conclusions drawn adequately supported by the results?

Partly

Are sufficient details of methods and analysis provided to allow replication by others?

Partly

Reviewer Expertise:

Psychophysics

I confirm that I have read this submission and believe that I have an appropriate level of expertise to confirm that it is of an acceptable scientific standard, however I have significant reservations, as outlined above.

# Reviewer report: Reviewer response for version 2

**DOI:** 10.5256/f1000research.176423.r368254

**Published:** 2025-02-28

**Authors:** Sandeep Maruthy

**Affiliations:** 1 Department of Audiology, All India Institute of Speech and Hearing, Mysuru, Karnataka, India

The authors have effectively addressed all the concerns I raised regarding version 1. I have noticed a significant improvement in version 2, with enhanced clarity and a more comprehensive approach. I recommend version 2 for acceptance.

Is the work clearly and accurately presented and does it cite the current literature?

Yes

If applicable, is the statistical analysis and its interpretation appropriate?

Yes

Are all the source data underlying the results available to ensure full reproducibility?

Yes

Is the study design appropriate and is the work technically sound?

Yes

Are the conclusions drawn adequately supported by the results?

Yes

Are sufficient details of methods and analysis provided to allow replication by others?

Yes

Reviewer Expertise:

Audiology

I confirm that I have read this submission and believe that I have an appropriate level of expertise to confirm that it is of an acceptable scientific standard.

# Reviewer report: Reviewer response for version 2

**DOI:** 10.5256/f1000research.176423.r358929

**Published:** 2025-02-05

**Authors:** Karen Banai

**Affiliations:** 1 University of Haifa, Haifa, Haifa District, Israel

Introduction: As detailed in the original review, the reason(s) for attempting auditory training are not clear. I suggested some relevant references in the original review

 Data analysis: The statistical model is still not described in full. What were the random effect(s)?

 Results:

 1) It is still not clear what the F values in Table 2 stand for (full model? interaction? one of the main effects)?

 2)Please provide full statistical details about the time x group interactions for all outcome measures since this is the only indication of effectiveness. If those are the F values reported in Table 2, please explain.

Is the work clearly and accurately presented and does it cite the current literature?

Partly

If applicable, is the statistical analysis and its interpretation appropriate?

Partly

Are all the source data underlying the results available to ensure full reproducibility?

Yes

Is the study design appropriate and is the work technically sound?

Partly

Are the conclusions drawn adequately supported by the results?

Partly

Are sufficient details of methods and analysis provided to allow replication by others?

Partly

Reviewer Expertise:

Cognitive hearing science; perceptual learning of speech.

I confirm that I have read this submission and believe that I have an appropriate level of expertise to confirm that it is of an acceptable scientific standard, however I have significant reservations, as outlined above.

# Reviewer report: Reviewer response for version 1

**DOI:** 10.5256/f1000research.167573.r332064

**Published:** 2024-12-24

**Authors:** Sandeep Maruthy

**Affiliations:** 1 Department of Audiology, All India Institute of Speech and Hearing, Mysuru, Karnataka, India

**Introduction**

 The introduction is well-written, with the authors providing a strong justification for the study by critically evaluating existing research in the field.

**Method**

 Rephrase: to 'The participants were randomly assigned to either the experimental group or the control group using a simple randomization method involving the drawing of chits.'

 Smriti Shravan Software Version 2 was developed by  (Kumar & Maruthy, 2013). Correct it. 

 post-training (after 15 days): after 15 days of what?

 follow-up (after one month): after 1 month of what?

 The authors' decision to use 8 sessions as the criterion for considering data for analysis should be justified.

 The time points at which the control group was tested should be defined

 The control group did not receive any training. In such a case how do you judge that auditory-cognitive training made the difference in the performance. It could be mere training of any form. Include explanation for this.

 Give an operational definition of '

 Auditory resolution measures'

 'ms' is a standard abbreviation for milliseconds. Consider using the abbreviation.

 The authors state that 'The step size was adjusted according to individual subjects.' - how was it adjusted? please provide more details. 

 In the sentence 'The task of detecting temporal modulation (MD) involved three carrier stimuli of broadband noise (BBN), each lasting 350 milliseconds, with an inter-stimulus interval (ISI) of 500 milliseconds.', it is not clear as to what is the task of the subject. Please rephrase. 

**Results**

 Figure 1 can be moved to the end of Method section. 

 It is appreciated that the subjects were randomly assigned to the control and experimental groups. However, it is recommended to statistically confirm that there is no significant difference between the two groups in the baseline assessment.

 It is stated that "All 22 participants completed the training or observation," which is incorrect. Only 11 participants underwent the training.

**Discussion**

 The discussion is well-written; however, using this research design makes it challenging for the authors to attribute the observed improvements solely to auditory cognitive training. This limitation will remain a caveat of the study.

 General comment: The manuscript would benefit from a more thorough proofreading to enhance its readability.

Is the work clearly and accurately presented and does it cite the current literature?

Yes

If applicable, is the statistical analysis and its interpretation appropriate?

Yes

Are all the source data underlying the results available to ensure full reproducibility?

Yes

Is the study design appropriate and is the work technically sound?

Yes

Are the conclusions drawn adequately supported by the results?

Yes

Are sufficient details of methods and analysis provided to allow replication by others?

Yes

Reviewer Expertise:

Audiology

I confirm that I have read this submission and believe that I have an appropriate level of expertise to confirm that it is of an acceptable scientific standard, however I have significant reservations, as outlined above.

# Response

**Published:** 2025-01-01

**Authors:** Gopee Krishnan

**Affiliations:** Speech and Hearing, Manipal Academy of Higher Education, Manipal, Karnataka, India

Revision 1:

**Reviewer Comments**

**1.Method**

 Rephrase: to 'The participants were randomly assigned to either the experimental group or the control group using a simple randomization method involving the drawing of chits.'

**Corrections: **Comments accepted and modified

**Location: **Methods section, Randomization and Blinding paragraph

 2.Smriti Shravan Software Version 2 was developed by (Kumar & Maruthy, 2013). Correct it. 

**Corrections: **Comments accepted and incorporated

**Location: **Methods section, Experimental group 1 ^st^ paragraph

 3.The authors' decision to use 8 sessions as the criterion for considering data for analysis should be justified.

**Clarification: **It was the minimum criterion set earlier for inclusion of data for analysis and to minimize loss of data due to attrition. All participants completed fifteen training sessions. Therefore, the phrase “8 sessions as the criterion for considering data for analysis” can be removed.

**Location: **Methods section, Experimental group 3 ^rd^ paragraph

 4.post-training (after 15 days): after 15 days of what?

 follow-up (after one month): after 1 month of what?

**Corrections: **Comments accepted and modified as [post-training (after 15 days of training)

 Follow-up (after one month of post-training assessment)]

**Location: **Methods section, Experimental group 3 ^rd^ paragraph

 5.The time points at which the control group was tested should be defined

**Corrections: **Suggestion accepted and incorporated.  Assessments were conducted for the control group at three-time points: baseline, post-observation (15 days after the baseline assessment), and follow-up observation (one month after the post-observation assessment).

**Location: **Methods section, Control group paragraph

 6.Give an operational definition of 'Auditory resolution measures'

**Corrections: **The auditory resolution measure refers to an individual's ability to detect the smallest variations in a given stimulus, such as variations in frequency (Hz) and time (dB or ms). 

**Location: **Methods section, Auditory resolution measures paragraph

 7.'ms' is a standard abbreviation for milliseconds. Consider using the abbreviation.

**Corrections: **Comments accepted and modified

**Location: **Auditory resolution measures, 2 ^nd^ paragraph

 8.The authors state that 'The step size was adjusted according to individual subjects.' - how was it adjusted? please provide more details. 

**Corrections: **The software adjusted the step size based on participant responses. It gradually increased the step size for incorrect responses and decreased it for correct responses.

**Location: **Auditory resolution measures, 2 ^nd^ paragraph

 9.In the sentence 'The task of detecting temporal modulation (MD) involved three carrier stimuli of broadband noise (BBN), each lasting 350 milliseconds, with an inter-stimulus interval (ISI) of 500 milliseconds.', it is not clear as to what is the task of the subject. Please rephrase. 

**Corrections: **Suggestion accepted and modified. “The temporal modulation task involved three carrier stimuli consisting of broadband noise (BBN). Each stimulus lasted for 350 milliseconds, and there was an inter-stimulus interval (ISI) of 500 milliseconds. Participants were instructed to identify the stimulus that was different (modulated) from the other two stimuli”.

**Location: **Auditory resolution measures, 3 ^rd^ paragraph

**10.Results**

 Figure 1 can be moved to the end of Method section. 

**Corrections: **Accepted and moved.

**Location: **Methods section

 11.It is stated that "All 22 participants completed the training or observation," which is incorrect. Only 11 participants underwent the training.

**Corrections: **All 11 participants in the experimental group completed the training, and their data was included in the analysis.

**Location: **Results section, 1 ^st^ paragraph

 12.It is appreciated that the subjects were randomly assigned to the control and experimental groups. However, it is recommended to statistically confirm that there is no significant difference between the two groups in the baseline assessment.

**Corrections: **Suggestion accepted.

 A table for Baseline Demographic Information is included below. The explanation related to the table is included in the text.

 “The statistical analysis indicated no significant differences in baseline assessments between the control and experimental groups for most outcome measures, except for TMT Part B-A”.

**Location: **Results section, 1 ^st^ paragraph

 Given the significant differences observed at baseline for TMT Part B-A, post-training significance should be interpreted cautiously before generalizing.

**Location: **Discussion section, 5 ^th^ paragraph

**13.Discussion**

 The discussion is well-written; however, using this research design makes it challenging for the authors to attribute the observed improvements solely to auditory cognitive training. This limitation will remain a caveat of the study.

**Corrections: **Thank you for the suggestion. This limitation is included in the revised manuscript.

 14.The control group did not receive any training. In such a case how do you judge that auditory-cognitive training made the difference in the performance. It could be mere training of any form. Include explanation for this.

**Corrections: **We agree that the difference in performance of the control group needs explanation. Including an active control group in this study would have eliminated the possibility of a placebo effect in the experimental group. Additionally, various factors, such as participant motivation and health condition on the day of assessment, could have influenced their performance. Furthermore, the literature supports the absence of evidence indicating differences between the active and passive control groups in cognitive interventions (Au et al., 2024).

**Location: **Discussion section, 7 ^th^ paragraph

 General comment: The manuscript would benefit from a more thorough proofreading to enhance its readability.

**Table 1 Baseline Demographic Information**

**Variables**

**Experimental group**

**(n=11) Mean (SD)**

**Control group**

**(n=11) Mean (SD)**

***p*** **-value**

 Age (years)

**EG: **70.4 (5.71)

**CG:** 68.0 (7.18)

**p-value:** 0.403

 MoCA

**EG: **22.2 (1.99)

**CG: **21.5 (2.16)

**p-value: **0.481

 FD

**EG:**129.36 (119.20)

**CG:** 56.45 (41.45)

**p-value: **0.070

 MD

**EG: **-13.75 (2.32)

**CG:** ​​​​​​​-14.02 (3.83)

**p-value: **0.847

 TGD

**EG: **22.71 (42.48)

**CG:** 9.65 (3.88)

**p-value: **0.322

 TMT Part A

**EG: **98.27 (46.29)

**CG:** 99.27 (29.15)

**p-value: **0.952

 TMT Part B

**EG: **289.73 (120.36)

**CG:** 217.73 (47.82)

**p-value: **0.080

 TMT Part B-A

**EG: **191.45 (80.71)

**CG:** 118.45 (33.09)

**p-value: **0.012*

 DR

**EG: **4.55 (3.45)

**CG:** 5.55 (2.30)

**p-value: **0.433

 *Level of Significance ( *p*<0.05)

# Reviewer report: Reviewer response for version 1

**DOI:** 10.5256/f1000research.167573.r329238

**Published:** 2024-10-25

**Authors:** Karen Banai

**Affiliations:** 1 University of Haifa, Haifa, Haifa District, Israel

In this preliminary study, the potential contribution of auditory-cognitive training to auditory processing and to performance on two cognitive tasks (recall and trail making) was studied using a RCT. Participants were 22 older adults with MCI randomized to an intervention and a control group. The intervention group participated in 15 1-hour training sessions focused on auditory span tasks using digits. All participants were tested on the auditory and cognitive outcome measures at three time points: pre- post- and follow-up. Some improvements in the outcome measures were observed over time , but it is unclear whether they can be consistently attributed to training.

 The study is generally well designed, and used tester-blinding to minimize researcher-related bias. Note that my review did not cover the inclusion criteria and the diagnosis of MCI. Comments on specific aspects of the study are listed below. These should be addressed to make the study sounder.

**General**

 1) Although are-related hearing loss and MCI often co-occur, no model of the potential causal associations is presented. Therefore, it is not clear why auditory memory training should have influenced auditory processing as assessed by the gap, frequency and modulation tasks.  

 2) On a related note, one of the motivations for this study was that only few previous studies delivered cognitive training via the auditory modality to people with MCI. However, if auditory processing is one of the contributing factors to the cognitive profile of people with MCI, why is it desirable to deliver training via the auditory modality?

 3) One recent systematic review on the effects of cognitive training in MCI is cited, but others are not (e.g., Kudlicka, A., Martyr, A., Bahar-Fuchs, A., Sabates, J., Woods, B., & Clare, L. (2023 [Ref - 1]). Cognitive rehabilitation for people with mild to moderate dementia. The Cochrane database of systematic reviews, 6(6), CD013388. https://doi.org/10.1002/14651858.CD013388.pub2).

**Methods**

 1) Age, hearing and MOCA are not reported separately for each group, making it hard to determine whether the randomization yielded well matched groups. This is a concern given some of the data reported in Table 1 which would suggest that the two groups may not have been well matched on the outcome measures at the onset of the study.

 2) It is not clear whether the same versions of the cognitive outcome measures were used across the pre- post- and follow-up tests or if parallel forms were used.

 3) Whereas the title and the abstract of the paper suggest that auditory working memory was targeted as an outcome measure, a verbal learning test is described in the methods, which has a working memory component, but may not be as clear index of working memory as other tests.

 4) Although testers during the pre- post- and follow-up tests were blind to whether participants received training or not, the use of an untrained group that received no alternative or sham intervention would make it hard to determine whether outcomes are a byproduct of the specific intervention or of participants having received any sort of intervention (see Jacoby, N., & Ahissar, M. (2013 [Ref - 2]). What does it take to show that a cognitive training procedure is useful? A critical evaluation. *Progress in brain research*, *207*, 121–140. https://doi.org/10.1016/B978-0-444-63327-9.00004-7).

 5) How were the training session distributed? Daily? Weekly? Some other schedule?

 6) What was the schedule for the pre- post- and follow-up tests? Did the two groups follow the same schedule?

**Data presentation and analysis**

 1) The statistical model used is not fully reported.

 (a) What were the random effects?

 (b) What kind of follow-up or planned comparisons were used to obtain the effects between test times that are reported with the a,b,c indices in Table 1?

 (c) It is not clear whether the F-values in Table 1 describe group, time or interaction effects.

 (d) Table 1 suggests that the significance in modulation detection may have been driven by pre- to post- changes in the control group. In the absence of a more detailed description of the statistical outcomes and tests, it is hard to justify the discussion statement that “The results revealed that AbCT positively impacted MD function...”.

Is the work clearly and accurately presented and does it cite the current literature?

Partly

If applicable, is the statistical analysis and its interpretation appropriate?

Partly

Are all the source data underlying the results available to ensure full reproducibility?

Yes

Is the study design appropriate and is the work technically sound?

Partly

Are the conclusions drawn adequately supported by the results?

Partly

Are sufficient details of methods and analysis provided to allow replication by others?

Partly

Reviewer Expertise:

Cognitive hearing science; perceptual learning of speech.

I confirm that I have read this submission and believe that I have an appropriate level of expertise to state that I do not consider it to be of an acceptable scientific standard, for reasons outlined above.

## References

[1] : Cognitive rehabilitation for people with mild to moderate dementia. *Cochrane Database Syst Rev* .2023;6(6) : 10.1002/14651858.CD013388.pub2 CD013388 10.1002/14651858.CD013388.pub2 37389428 PMC10310315

[2] : What does it take to show that a cognitive training procedure is useful? A critical evaluation. *Prog Brain Res* .2013;207: 10.1016/B978-0-444-63327-9.00004-7 121-40 10.1016/B978-0-444-63327-9.00004-7 24309253
